# Cost-Effectiveness of Breast Cancer Control Strategies in Central America: The Cases of Costa Rica and Mexico

**DOI:** 10.1371/journal.pone.0095836

**Published:** 2014-04-25

**Authors:** Laurens M. Niëns, Sten G. Zelle, Cristina Gutiérrez-Delgado, Gustavo Rivera Peña, Blanca Rosa Hidalgo Balarezo, Erick Rodriguez Steller, Frans F. H. Rutten

**Affiliations:** 1 Institute for Medical Technology Assessment and Institute for Health Policy & Management, Erasmus University Rotterdam, Rotterdam, The Netherlands; 2 Department of Primary and Community Care, Radboud University Nijmegen Medical Center, Nijmegen, The Netherlands; 3 Instituto Mexicano del Seguro Social (IMSS), México DF, México; 4 Unidad de Análisis Económico, Secretaria de Salud, México D.F., México; 5 Pharmaco-economics department Caja Costarricense de Seguro Social, San José, Costa Rica; 6 Ministerio de Salud, San José, Costa Rica; Endocrine Research Center (Firouzgar), Institute of Endocrinology and Metabolism, Iran (Islamic Republic of)

## Abstract

This paper reports the most cost-effective policy options to support and improve breast cancer control in Costa Rica and Mexico. Total costs and effects of breast cancer interventions were estimated using the health care perspective and WHO-CHOICE methodology. Effects were measured in disability-adjusted life years (DALYs) averted. Costs were assessed in 2009 United States Dollars (US$). To the extent available, analyses were based on locally obtained data. In Costa Rica, the current strategy of treating breast cancer in stages I to IV at a 80% coverage level seems to be the most cost-effective with an incremental cost-effectiveness ratio (ICER) of US$4,739 per DALY averted. At a coverage level of 95%, biennial clinical breast examination (CBE) screening could improve Costa Rica's population health twofold, and can still be considered very cost-effective (ICER US$5,964/DALY). For Mexico, our results indicate that at 95% coverage a mass-media awareness raising program (MAR) could be the most cost-effective (ICER US$5,021/DALY). If more resources are available in Mexico, biennial mammography screening for women 50–70 yrs (ICER US$12,718/DALY), adding trastuzumab (ICER US$13,994/DALY) or screening women 40–70 yrs biennially plus trastuzumab (ICER US$17,115/DALY) are less cost-effective options. We recommend both Costa Rica and Mexico to engage in MAR, CBE or mammography screening programs, depending on their budget. The results of this study should be interpreted with caution however, as the evidence on the intervention effectiveness is uncertain. Also, these programs require several organizational, budgetary and human resources, and the accessibility of breast cancer diagnostic, referral, treatment and palliative care facilities should be improved simultaneously. A gradual implementation of early detection programs should give the respective Ministries of Health the time to negotiate the required budget, train the required human resources and understand possible socioeconomic barriers.

## Introduction

Due to population ageing and changing lifestyles in low-and-middle countries (LMICs), breast cancer incidence rates are increasing [1], [2]. Given the organizational and financial constraints faced by the health systems in LMICs the majority of breast cancers are diagnosed at late stages [3]. Accordingly, the majority of breast cancer deaths occur in LMICs [4], [5]. The World Health Organization (WHO) therefore states that early detection and implementation of cost-effective interventions should be a priority in LMICs [6]. In an attempt to support LMICs with breast cancer control, the Susan G. Komen for the cure foundation provided a grant to investigate the cost-effectiveness of several breast cancer control interventions in 7 LMICs (Brazil, Colombia, Costa-Rica, Ghana, India, Mexico and Peru) to a consortium of the WHO, Erasmus University Rotterdam (EUR) and Radboud University Nijmegen Medical Center (RUNMC). Cost-effectiveness analyses may support governments in deciding how to spend scarce resources in health care most efficiently.

In each country, during four phases, the consortium works closely with local authorities and experts in the fields of breast cancer, health economics, epidemiology and public policy. First, a three-day technical workshop is held, where the consortium explains a general cost-effectiveness model based on WHO-CHOICE methodology (described elsewhere [7], [8]) which is to be tailored to the country specific situation. In the second phase, lasting approximately six months, local authorities identify and assemble the (local) data required for the cost-effectiveness model. Subsequent in phase three, the cost-effectiveness analyses are carried out. Thereafter, a second workshop is organized. Here the results of the analyses are discussed among representatives of all local institutions involved in breast cancer care and made available for actual policy making by the local health authorities, i.e. the fourth phase. This paper identifies the most cost-effective interventions for breast cancer control in both Costa Rica and Mexico from a health care perspective.

After presenting an overview of the situation regarding breast cancer in both Costa Rica and Mexico, we discuss the methods, data and interventions considered in this study and discuss the results.

### Breast cancer in Costa Rica and Mexico

Cancer incidence and mortality rates are rising across Central America [9], [10]. In Costa Rica and Mexico breast cancer ranks among the top-five causes of death for women over 25 years old [11]. Between 1995 and 2003, breast cancer incidence increased by 32.3% to a rate of 40.07 per 100,000 women in Costa Rica [12]. In Mexico, breast cancer incidence increased as well and in both countries breast cancer mortality rates have increased since the 1980s [9], [13], [14]. In Costa Rica 13.14 breast cancer deaths per 100,000 women in 2006, the highest number among malignant neoplasms, are observed. Mortality rates per 100,000 women range from 28.19 in province ‘Dota’ to 1.23 Guácimo, while in provinces ‘Los Chiles, ‘La Cruz’, and ‘Garabito’ no breast cancer related deaths were registered [12]. In Mexico mortality rates doubled over the last 20 years. The average mortality rate per 100,000 women in Mexico stands at 9.9 with regional differences from 13.2 and 11.8 respectively in the Federal District and the north to 9.7 and 7.0 respectively in the center and the south [15]. This increase caused breast cancer to overtake cervical cancer as the most deadly cancer among females in 2006 [14], [15].Where in 1979 1,144 females died from the disease, in 2006 4,497 deaths were registered [15].

Although in Costa Rica and Mexico official recommendations for both breast self-examination (BSE) and mammography screening have existed for over a decade, their coverage levels remain very low and the large majority of breast cancer patients present at the hospital with advanced disease [16]–[18].

In light of the above, Non-Governmental Organizations (NGOs) and the general public put pressure on governments in Costa Rica and Mexico to improve treatment and early diagnosis through screening [19], [20]. Hence, both countries face choices on efficient allocation of scarce resources for breast cancer screening.

## Materials and Methods

### Methods

#### General approach

We used the WHO-CHOICE methodology, described in detail elsewhere [7], [8], as a basis of our analysis. This approach compares all possible interventions in a specific disease area to a situation where no interventions are implemented. The latter, a counterfactual ‘null scenario’, acts as a reference to compare the costs and effects of existing and new interventions. An intervention in isolation, or a combination of different interventions, is then implemented for 10 years in a modeled population. However, to include effects that occur after these 10 years, this modeled-population is tracked for 100 years. This approach enables us to make comparisons of the costs and health effects across a wide range of competing interventions, identify differences in relative cost-effectiveness and identify the most efficient mix of interventions to improve population health.

#### Breast Cancer Model

**Figure 1:**
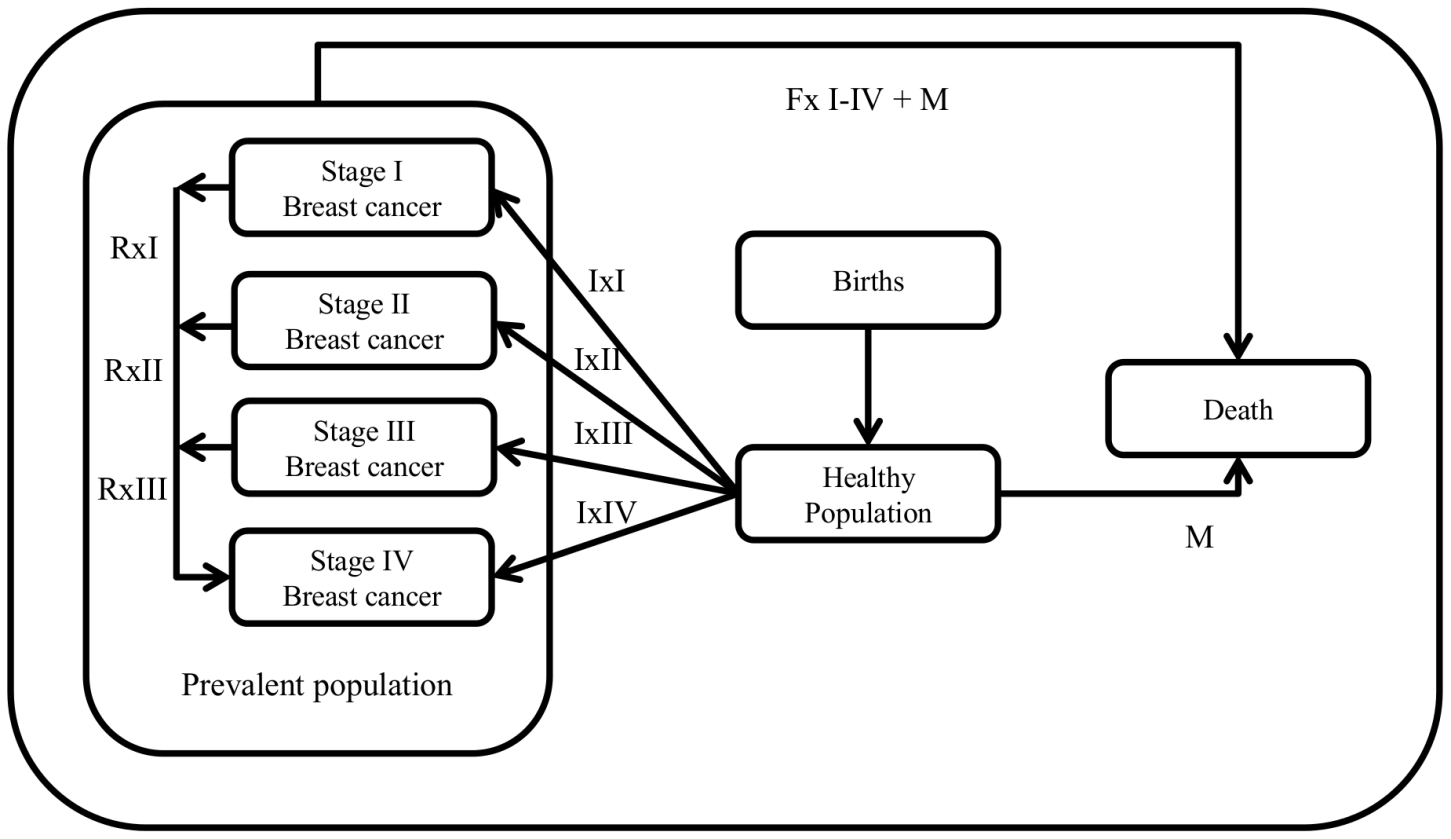
Graphical representation of the model showing the relationships between the different health states through the incidence rates of breast cancer (Ix1–Ix4), the different stage specific case fatality rates (Fx1–4), and the background mortality (M) [7]. Stage specific relapse rates to stage IV were used to correct health state valuations only (Rx1–Rx3).

Costs and health effects are calculated using a state transition population model developed and explained in detail by Groot et al. [7]. Its structure is presented in Figure 1 [7]. The model simulates the development of a national population and accounts for births, background mortality and breast cancer epidemiology of a country. It includes a healthy state, a deceased state, and stage I to IV breast cancer states following the classification of the American Joint Committee on Cancer (AJCC) [21]. The effectiveness of each intervention is expressed in changes in disability weights (DWs i.e. health state valuations (HSVs)), case fatality rates (CFs, i.e. improved survival for treatment scenarios), or in more positive stage distributions (in awareness raising and screening interventions). Since the interventions affect mortality (i.e., CFs) and morbidity (DWs), intervention effectiveness is expressed in disability adjusted life years (DALYs) averted. The difference in the total number of healthy years lived by the population between each scenario and the null-scenario gives the population health gains in DALYs averted.Zelle et al. [22] improved the published model [7] by correcting HSVs for relapse, assuming that patients could only relapse to stage IV at a constant rate [23].

#### Interventions

An important element of the overall project is to select a set of appropriate interventions for breast cancer control in LMICs. Therefore, a study group at WHO-CHOICE defined a mix of 11 common and preferable practices in 2009 [22]. Participating countries can combine and adapt these practices to appropriately inform their specific policy questions. For Costa Rica and Mexico focus was placed on the cost-effectiveness of screening & treatment combinations. The most urgent policy questions in both countries concerned the age groups that should be targeted for screening and whether treating Her2/NEU+ patients with Trastuzumab was cost-effective. Therefore, the basic awareness raising intervention was excluded and different intervention scenarios, including treatment with Trastuzumab, were added. Combining the 11 common practices with or without Trastuzumab led to a total of 19 scenarios. Input from local policy makers led us to model the current situations of breast cancer control in Costa Rica and Mexico at 80% and 70% geographic coverage levels (i.e. reaching 80%/70% of those people who need services) respectively. In line with WHO-CHOICE methodology all other interventions were evaluated at a geographic coverage level of 95% [8]. An overview of the interventions is listed in table 1.

**Table 1:** Definition and classification of individual interventions (coverage) (based on [22]).

Treatment of individual stages	Down-staging interventions^b^	Palliative care^d^
Stage I treatment: lumpectomy withaxillary dissection and radiotherapy. Eligible patients receive tamoxifen^a^ or chemotherapy^e^ [7], [23], [49].	Basic Awareness Raising (BAR): community nurses training program+opportunistic outreach activities by community nurses to raise breast cancer awareness and educate on breast self-examination techniques (BSE)+enhanced media activities [50].^c^	Basic Palliative Care (BPC): palliative care volunteers training program+home-based visits by volunteers every fortnight+pain treatment through morphine, laxatives and palliative radiotherapy (8 Gy in 1 fraction) for eligible patients [49]–[51]
Stage II treatment: lumpectomy with axillary dissection and radiotherapy. Eligible patients receive tamoxifen^a^ or Chemotherapy^e^ [7], [23], [49].	Mass-media awareness raising (MAR): BAR+mass media campaign [50].	Extended Palliative Care (EPC): BPC apart from community nurses instead of palliative care volunteers, pain treatment strengthened with antidepressants, anti-emetics and zelodronic acid [50]–[54].
Stage III treatment: modifiedmastectomy followed by adjuvantchemotherapy^e^ and radiotherapy^f^.Eligible patients receive tamoxifen^a^ [7], [49].	Biennial clinical breast examination (CBE) screening in asymptomatically women aged 40–69 years: community nurses training program+active outreach screening by community nurses+limited media activities [50], [55].	
Stage IV treatment: adjuvant Chemotherapy^e^ and radiotherapy (10 Gy)+end of life hospitalization. Eligible patients receive total mastectomy and/or tamoxifen^a^ [49], [56].	Biennial mammography screening in asymptomatic women aged 50–69 years+limited media activities [7].	
Treatment of stage I–IV as listed above plus the addition of Trastuzumab^g^ for Her2/NEU+ patients.	Biennial mammography screening in asymptomatic women aged 40–69 years+limited media activities [7].^c^	

a Endocrine therapy consists of 20 mg tamoxifen per day for 5 years.

b Down-staging interventions cause a shift in stage distribution and are only modeled in combination with treatment of all stages (I–IV).

c BAR was excluded as a standalone intervention in Costa Rica and Mexico.

d Palliative care interventions are only applied to stage IV patients, and substitutes stage IV treatment.

e The (neo)adjuvant chemotherapy combination regimen consists of 7 cycles of Epirubicin, Fluorouracil and cyclophosphamide (FEC regimen) Given on an outpatient basis.

f Radiotherapy includes a standard dose of 50 Gy given in 25 fractions of 2 Gy on an outpatient basis.

g Trastuzumab is given for 8 months.

### Data

#### Effectiveness

A key factor is the stage distribution of patients presenting at the hospital, given the breast cancer stage determines the survival and disability of the breast cancer patients and the effectiveness of each intervention [21].

In Costa Rica we obtained the current stage distribution from Ortiz [24], who studied breast cancer survival in Costa Rica between 2000 and 2003. Demographic data and incidence rates were obtained from the Statistical office of the Costa Rican Ministry of Health (MoH). For the prevalence we used the 2004 Global Burden of Disease estimates [25].

For Mexico, we used the current stage distribution from Knaul et al. [17], who studied 1904 patients that were all treated within the Mexican Social Security Institute (IMSS, its abbreviation in Spanish). Demographic data were obtained from the Mexican National Population Council [29]. For Mexico we obtained incidence rates based on GLOBOCAN 2008 adjusted by group of age considering the distribution from the Mexican Histopathology Registry 2006 [30], [31]. For the prevalence in Mexico, as in Costa Rica, we used 2004 Global Burden of Disease estimates [25].

The case fatality rates for the treatment scenarios were based on Groot et al. (stage III & IV) and Zelle et al. (stage I & II), who corrected those from Groot et al. for the use of chemotherapy in stage I and II [7], [22]. We take these CF's to represent technical efficiency, representing the maximum amount of DALYs that can be averted based on successful implementation of breast cancer diagnosis, treatment and follow-up. Disability weights were derived from the Global Burden of Disease estimates for long term sequela [25] using quality of life literature [26], [27]. For stage I we took the disability estimate of 0.086 [28] and for stage IV we combined the terminal estimate of 0.75 [28] with estimates from quality of life literature [26].

Since screening and awareness interventions as defined in international literature, alter the stage distribution, their effects on the stage distribution at presentation were estimated using the same methods applied by Zelle et al. [22]. Zelle et al. [22] use international study results to estimate the health effects of various screening options and account for the sensitivity of the screening method, attendance rates (80% in both countries), incidence rates and demography in target groups. For each intervention, table 2 gives an overview of the estimates used in every stage.

**Table 2:** Analyzed interventions and the estimates used for the stage were interventions are applied to.

	CF Rates^a^	CF Rates^a^	CF Rates^a^	CF Rates^a^	DW's^b^	DW's^b^	DW's^b^	DW's	Stage Dist.^c^	Stage Dist.^c^	Stage Dist.^c^	Stage Dist.^c^
Costa Rica (CR) - Intervention	stage I	Stage II	Stage III	Stage IV	stage I	Stage II	Stage III	Stage IV	% in stage I	% in stage II	% in stage III	% in stage IV
Untreated	0.0207	0.0654	0.1556	0.3112	0.086	0.097	0.104	0.375	14.6%	41.6%	20.4%	23.4%
Stage I treatment	0.0056				0.086				14.6%			
Stage II treatment		0.0393				0.097				41.6%		
Stage III treatment			0.0930				0.104				20.4%	
Stage IV treatment				0.2750				0.154				23.4%
Basic Palliative Care (BPC)				0.2750				0.153				23.4%
Extended Palliative Care (EPC)				0.2750				0.152				23.4%
Current Country Situation	0.0056	0.0393	0.0930	0.2750	0.086	0.097	0.104	0.154	14.6%	41.6%	20.4%	23.4%
Mass-media Awareness Raising (MAR)	0.0056	0.0393	0.0930	0.2750	0.086	0.097	0.104	0.154	21.1%	41.5%	24.1%	13.3%
Biennial CBE screening (40–69)	0.0056	0.0393	0.0930	0.2750	0.086	0.097	0.104	0.154	32.0%	34.3%	25.8%	7.9%
Biennial mammography screening (50–69)	0.0056	0.0393	0.0930	0.2750	0.086	0.097	0.104	0.154	35.0%	37.5%	21.1%	6.5%
Biennial mammography screening (40–69)	0.0056	0.0393	0.0930	0.2750	0.086	0.097	0.104	0.154	40.0%	42.8%	13.2%	4.0%
With Trastuzumab	0.0050	0.0353	0.0835	0.2470	0.086	0.097	0.104	0.154				

Mexico(MX) - Intervention	stage I	Stage II	Stage III	Stage IV	stage I	Stage II	Stage III	Stage IV	% in stage I	% in stage II	% in stage III	% in stage IV
Untreated	0.0207	0.0654	0.1556	0.3112	0.086	0.097	0.104	0.375	13.8%	39.6%	33.9%	12.7%
Stage I treatment	0.0056				0.086				13.8%			
Stage II treatment		0.0393				0.097				39.6%		
Stage III treatment			0.0930				0.104				33.9%	
Stage IV treatment				0.2750				0.154				12.7%
Basic Palliative Care (BPC)				0.2750				0.153				12.7%
Extended Palliative Care (EPC)				0.2750				0.152				12.7%
Current Country Situation	0.0056	0.0393	0.0930	0.2750	0.086	0.097	0.104	0.154	13.8%	39.6%	33.9%	12.7%
Mass-media Awareness Raising (MAR)	0.0056	0.0393	0.0930	0.2750	0.086	0.097	0.104	0.154	21.1%	41.5%	24.7%	12.7%^d^
Biennial CBE screening (40–69)	0.0056	0.0393	0.0930	0.2750	0.086	0.097	0.104	0.154	30.5%	32.6%	28.3%	8.7%
Biennial mammography screening (50–69)	0.0056	0.0393	0.0930	0.2750	0.086	0.097	0.104	0.154	33.9%	36.3%	22.8%	7.0%
Biennial mammography screening (40–69)	0.0056	0.0393	0.0930	0.2750	0.086	0.097	0.104	0.154	39.1%	41.8%	14.6%	4.5%
With Trastuzumab	0.0054	0.0374	0.0865	0.2569	0.086	0.097	0.104	0.154				

a Case Fatality - Estimates for stages III and IV are from Groot et al. [7] and for stages I and II from Zelle et al. [22]. The CFs for the untreated patients are from Groot et al. [7] and were corrected based on Bloom et al [57].

b Disability Weights - Estimates from Zelle et al.[22].

c Current stage distribution CR is based on Ortiz [24]; MX onKnaul et al. [17]; Effects of MAR derived from Devi [50]; Effects of screening interventions were based on stage shifts from baseline Groot et al.[7] to the stage distribution USA in Bland et al. [58]. Stage shifts were adapted by calculating relative differences in detection rates between the USA and CR/MX from Duffy & Gabe [59]. Calculations included age-specific incidence (MoH CR & Unidad Analysis Económica MX), prevalence (WHO 2008), sojourn time Duffy & Gabe [59], sensitivity Bobo et al. [60] and attendance rates (75% in the USA vs. 80% in Costa Rica and Mexico).

d We assumed in Mexico implementing MAR could not lead to a higher proportion of stage IV patients and increase stage III with the difference of 0.6%.

#### Costs

In line with the WHO-CHOICE approach we distinguished patient, program and training costs, which were calculated by multiplying quantities of applied procedures by their corresponding unit costs. Patient costs were dependent on patient consumption (utilization) of explicit resources (procedures) for diagnosis, treatment, follow-up, early detection and screening.

Although Costa Rica has developed several guidelines for treating breast cancer over the years [18], [32], local specialists informed us that treatments differ somewhat across hospitals. Therefore, together with these specialists, we revised the entire set of resource items to reflect the (average) current breast cancer treatment practices in Costa Rica.

In Mexico the health care system has three main public institutions providing health care to different groups. Whereas the Instituto Mexicano del Seguro Social (IMSS) covers salaried workers in the private sector, the Instituto de Seguridad y Servicios Sociales de los Trabajadores del Estado (ISSSTE) provides benefits for government workers. Finally, Seguro Popular concerns voluntary public insurance for the non-salaried workers or unemployed. Specialists in Mexico informed us treatment and reimbursement between these institutions may differ due to, for example, differences in salaries and drug prices. Hence we used resource utilization estimates of IMSS, which provides social insurance to approximately 40% of Mexico's population [33].

Whenever possible we used locally obtained costing data. When not available we applied the original WHO-CHOICE estimates for either country. These estimates are based on econometric analysis of a detailed WHO-CHOICE database from South Africa including a set of standard salaries, drugs, outpatient visits, materials and supplies, capacity utilization and transportation multipliers [34]. In Costa Rica, the CCSS provided readily available unit costs of most breast cancer procedures. For Mexico, contrary to Salomon et al. [35], who used the WHO-CHOICE original estimates on costs, in this study we used a detailed micro-costing exercise performed by IMSS [36].

Costs of the procedures used for Costa Rica and Mexico are listed in table 3.We also integrated evaluation costs of women presenting without breast cancer, included the costs of diagnosing all other stages (only for stages I–IV separately) and, regarding screening interventions, included costs for evaluating false positives.

**Table 3:** Average utilization of diagnosis and treatment ingredients and unit costs per patient.

Procedure and Ingredients	Stage I	Stage I	Stage II	Stage II	Stage III	Stage III	Stage IV	Stage IV	Relapse	Relapse	Palliative Care^g^ (Extended)	Palliative Care^g^ (Extended)	Unit cost per patient (US$)	Unit cost per patient (US$)
Initial diagnosis and evaluation during treatment	Costa Rica	Mexico	Costa Rica	Mexico	Costa Rica	Mexico	Costa Rica	Mexico	Costa Rica	Mexico	Costa Rica	Mexico	Costa Rica	Mexico
No. of health center visits	1	1	1	1	1	1	1	1	1	1			23,69^a^	25,40^c^
No. of hospital visits	3	2	3	2	3	2	3	2	3	2			63,187^a^	80,47^c^
Bilateral Mammography	1	1	1	1	2	1	-	-	-	-			45,44^a^	42,27^d^
Complete blood count	7	7	7	7	7	7	7	7	6	6			17,50^b^	10,34^d^
FNA or core needle biopsy	1	1	1	1	1	1	1	1	-	-			71,62^a^	91,52^c^
Liver function tests	8	8	8	8	8	8	8	8	7	7			40,31^a^	10,34^d^
Ultrasonography	1	1	1	1	1	1	1	1	-	-			23,65^b^	48,32^d^
Renal function tests	8	8	8	8	8	8	8	8	7	7			9,81^a^	10,34^d^
Bone scan	-	-	-	-	1	1	1	1	-	-			108,01^b^	192,57^d^
Chest X-ray	1	1	1	1	1	1	1	1	-	-			16,11^a^	14,93^c^
ECG	1	1	1	1	1	1	1	1	-	-			10,14^b^	27,26^f^
Her2/neu test	1	1	1	1	1	1	1	1	-	-			27,73^e^	32,70^d^
**Non-breast cancer evaluation**														
No. of health center visits	2	2	2	2	2	2	2	2					23,69^a^	25,40^c^
Bilateral Mammography	1	1	1	1	1	1	1	1					45,44^a^	42,27^d^
Ultrasonography	0.28	0.28	0.28	0.28	0.28	0.28	0.28	0.28					22,68^b^	22,59^c^
FNA or core needle biopsy	0.02	0.02	0.02	0.02	0.02	0.02	0.02	0.02					71,62^b^	91,52^c^
**Treatment**														
No. of hospitalization days	2	2	2	2	2	2	6	0	6	0	6		134,55^a^	292,11^c^
No. of OPD visits radiotherapy	30	0	30	0	30	0	30	0	30	0	1	0	63,16^a^	80,47^c^
No. of OPD visits chemotherapy % receiving surgical intervention (Lump. = Lumpectomy and Mast. = Mastectomy)	6	7	6	7	6	7	6	7	6	7	-		63,16^a^	80,47^c^
	Lump.	Lump.	Lump.	Lump.	Lump.	Lump.	Lump.	Lump.	Lump.	Lump.	Lump.	Lump.	239,33^b^	805,59^c^
	60%	80%	60%	0,40%	20%	0%	-	-	-	-	-	-		
	Mast.	Mast.	Mast.	Mast.	Mast.	Mast.	Mast.	Mast.	Mast.	Mast.	Mast.	Mast.	243,27^b^	857,34^d^
	40%	20%	40%	60%	80%	30%	10%	-	10%	-	5%	-		
% receiving anesthesia	60%		70%		90%		5%	-	5%	-	5%	-	61,22^b^	76,68^c^
% receiving radiotherapy^h^	70%	86%	70%	80%	100%	100%	30%	0%	30%	0%	-	-	500,52^b^	438,20^c^
% receiving endocrine treatment^i^	61%	50%	61%	40%	61%	65%	61%	40%	61%	40%	61%	50%	0,04/day^a^	0,51^d^
% receiving chemotherapy^j^	0%	80%	20%	100%	60%	100%	60%	90%	80%	0%	-		1469,97^a^	2327,20^c^
% receiving boost radiotherapy^k^											41%	65%	71,23^b^	106,16^c^
% receiving home based visits											75%	75%	23,69^a^	25,40^c^
% receiving morphine^l^											84%	100%	0,59/day^a^	1,12^c^
% receiving laxative^m^											50%	47%	0,10/day^a^	0,03^c^
% receiving Ondansetron^n^											36%	60%	2,80/day^a^	1,72^c^
% receiving Amitriptyline^o^											41%	100%	0,04^a^	0,37^c^
% receiving Zelodronic Acid^p^							30%	30%	30%	30%	30%		200,00^a^	260,18^d^
% receiving Trastuzumab	30%	11%	30%	14%	30%	21%	30%	19%	30%	7%			1800^a^	1610^c^

a Based on estimates by Costa Rican CCSS.

b Unit costs WHO-CHOICE database in 2000 US$. Corrected for inflation: 2000–2009 (2.81 in CR & 1.66 in MX). 2009 exchange rates were used (560.45 CRC/US$ & 13.06 MXN/US$).

c Based on values of IMSS.

d Based on communication with Unidad de Análisis Económico of MoH.

e Based on Norum et al. [61].

f Based on Knaul et al. [11].

g palliative care (substitutes stage IV treatment).

h 50 Gy given in 25 fractions of 2 Gy.

i daily dose of 20 mg. Tamoxifen for 5 years.

j 7 cycles of Epirubicin, Fluorouracil and cyclophosphamide (FEC regimen).

k 1 fraction of 10 Gy.

l 40 ml/54 s days.

m 35 mg/54 days.

n 8 mg/day.

o 751mg/day.

p 5 mg/day.

For the program-level costs, which capture management, administrative, media and law-enforcement costs, and costs for training of healthcare personnel we used local salaries and WHO-CHOICE allocation rules for Costa Rica. For Mexico we used the standard WHO-CHOICE program cost estimates and allocation rules. Media and operating costs (i.e. prices for broadcasting, flyers, and posters) were provided by the CCSS in Costa Rica and the MoH in Mexico.

Training costs were primarily based on training the required health care workers for each intervention. We maintained the allocation assumptions listed in the WHO-CHOICE model as set by Zelle et al. [22] and used local salaries and WHO standard salaries for Costa Rica and Mexico respectively. In both countries all costs were estimated in 2009 local currency units (i.e. Costa Rican colones (CRC) and Mexican pesos (MXN)) and converted to U.S. dollars (US$) using the 2009 exchange rate (1.00US$ = 560.45CRC and 1.00US$ = 13.06MXN$) [34], [35]. Both health effects (DALYs) and costs (US$) were discounted at a rate of 3% annually, which is recommended by WHO-CHOICE [8]. Working from a health care perspective we did not take into account travel and opportunity costs.

#### Cost-effectiveness analysis

The average cost-effectiveness ratio (ACER) of each intervention is calculated by dividing the average costs of the intervention by average number of DALYs averted. These ACERs provide information on the set of interventions a region should finance to maximize health gains. The incremental cost-effectiveness ratios (ICERs) are calculated in relation to the last intervention purchased in each country, by dividing the incremental costs by the incremental health effects. These ICERs are used to establish an expansion path which shows the order in which the various interventions should be introduced if cost-effectiveness is the only consideration [39]. Only interventions with the lowest cost for additional effects are considered on this expansion path. WHO-CHOICE defines interventions that have a cost-effectiveness ratio of less than one times the gross domestic product (GDP) per capita as very cost-effective, and those with a ratio that falls between one and three times the GDP per capita as cost-effective [40]. In Costa Rica, this means that interventions that cost less than US$6,629 per DALY averted can be considered very cost-effective, and interventions that cost between US$6,629 and US$19,888 per DALY averted can be considered cost-effective. For Mexico these thresholds are US$8,416 and US$25,249 per DALY averted, respectively.

#### Sensitivity analysis

In line with Zelle et al. we performed a deterministic sensitivity analysis for both Costa Rica and Mexico to assess the impact of key parameters on our cost-effectiveness estimates [22]. In both countries we increased the DW's with 10%. Whereas costs of outpatient visits were increased by 25%, we raised the costs of mammography with 200%. In estimating the impact of various screening interventions we decreased the sensitivity of CBE and mammography by 25% and assumed attendance rates of screening of 60%. When available we also used alternative stage distributions for the current situation and different CFs. The unit costs for surgical procedures Costa Rica were much lower than those of Mexico. To test the impact of this we substituted these costs with the Mexican values.

## Results

**Figure 2:**
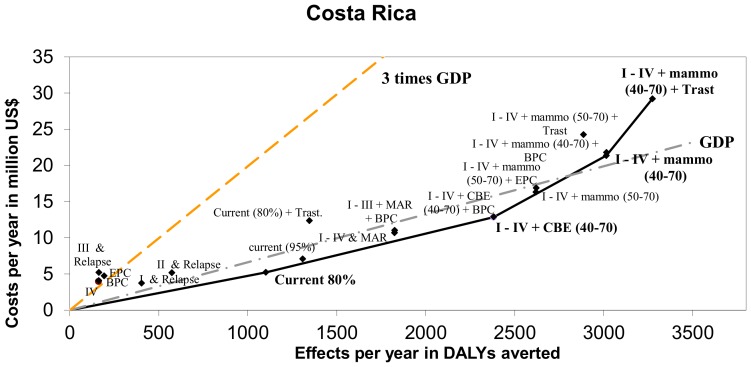
Cost-effectiveness of breast cancer interventions and expansion path according to Incremental Cost-Effectiveness Ratios for Costa Rica. Dotted lines represent cost-effectiveness threshold of 1 and 3 times 2009 GDP/capita, i.e. 6,629 US$/DALY and 19,888 US$/DALY [37], [38].

**Figure 3:**
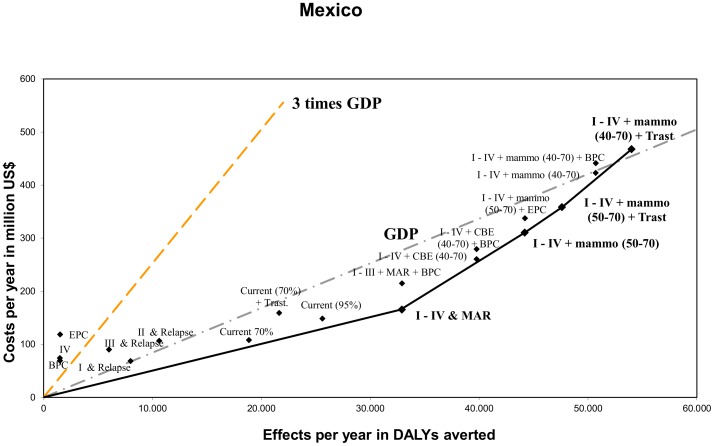
Cost-effectiveness of breast cancer interventions and expansion path according to Incremental Cost-Effectiveness Ratios for Mexico. Dotted lines represent cost-effectiveness threshold of 1 and 3 times 2009 GDP/capita, i.e. 8,416 US$/DALY and 25,249 US$/DALY [37], [38].

Tables 4 and 5 show the results for respectively Costa Rica and Mexico. Both costs, effects and cost-effectiveness are presented. In Figures 2 and 3 these results are presented graphically and the expansion paths are shown as black lines.

**Table 4:** Costa Rica - Average costs (US$), effects and cost-effectiveness of breast cancer control scenarios per year.

#	Description of intervention	Patients per year	Annual patient costs^a^	Annual program costs	Annual training costs	Annual total costs	DALYs averted per year^b^	ACER^c^	ICER^d^
1	Current country specific situation (80%)	940	4,569,310	646,358	6,660	5,222,329	1,102	4,739	4,739
2	Stage I to IV treatment (current)+Trastuzumab (80%)	940	11,708,670	646,358	6,660	12,361,689	1,347	9,180	NA
3	Stage I treatment+relapse (95%)	163	2,862,111	854,431	7,439	3,723,980	404	9,218	NA
4	Stage II treatment+relapse (95%)	464	4,303,195	854,431	7,439	5,165,065	573	9,007	NA
5	Stage III treatment+relapse (95%)	235	3,884,520	854,431	7,439	4,746,390	193	24,587	NA
6	Stage IV treatment (95%)	261	3,107,345	854,431	7,439	3,969,215	162	24,559	NA
7	Basic Palliative Care (BPC) (95%)	261	2,466,328	1,583,922	27,897	4,078,147	163	25,078	NA
8	Extended Palliative Care (EPC) (95%)	261	3,160,703	2,022,956	27,897	5,211,556	164	31,852	NA
9	Stage I to IV treatment combined (current 95%)	1,116	5,659,297	1,421,412	7,439	7,088,148	1,309	5,417	NA
10	Biennial mammography screening (50–70)+treatment of stage I to IV (95%)	1,116	12,498,059	3,792,653	22,317	16,313,029	2,619	6,228	NA
11	Biennial mammography screening (50–70)+treatment of stage I to IV+Trastuzumab (95%)	1,116	20,438,042	3,792,653	22,317	24,253,012	2,886	8,402	NA
12	Biennial mammography screening (40–70)+treatment of stage I to IV (95%)	1,116	17,546,792	3,792,522	22,317	21,361,632	3,015	7,085	13,426
13	Biennial mammography screening (40–70)+treatment of stage I to IV+Trastuzumab (95%)	1,116	25,401,093	3,792,522	22,317	29,215,932	3,274	8,924	30,352
14	Basic awareness outreach program+Mass-media Awareness Raising (MAR)+treatment of stage I to IV (95%)	1,116	6,158,209	4,519,154	11,159	10,688,521	1,825	5,857	NA
15	Biennial Clinical Breast Examination (CBE) screening (40–70)+treatment of stage I to IV (95%)	1,116	9,255,065	3,576,629	20,086	12,851,779	2,381	5,397	5,964
16	MAR+BPC+treatment of stage I to III (95%)	1,116	6,262,398	4,733,109	39,055	11,034,563	1,826	6,044	NA
17	Biennial CBE Screening+BPC+treatment of stage I to III (95%)	1,116	9,422,391	3,426,610	47,982	12,896,984	2,382	5,415	NA
18	Biennial mammography Screening (40–70)+BPC+treatment stage I to III (95%)	1,116	17,578,700	4,170,935	50,214	21,799,850	3,016	7,229	NA
19	Biennial mammography Screening (50–70)+EPC+treatment of stage I to III (95%)	1,116	12,620,626	4,215,537	50,214	16,886,376	2,621	6,444	NA

a All costs in this table are in 2009 US$ (1CRC = 0,001784 US$).

b DALYs, disability-adjusted life-years (age weighted, discounted).

c ACER: Average cost-effectiveness ratio compared to the do nothing-scenario (US$ per DALY averted).

d ICER: Incremental cost effectiveness ratio, ratio of additional cost per additional life-year saved when next intervention is added to a mix on the intervention path (additional US$ per additional DALY saved).

**Table 5:** Mexico - Average costs (US$), effects and cost-effectiveness of breast cancer control scenarios per year.

#	Description of intervention (coverage level)	Patients per year	Annual patient costs^a^	Annual program costs	Annual training costs	Annual total costs	DALYs averted per year^b^	ACER^c^	ICER^d^
1	Current country specific situation (70%)	12,682	105,806,655	2,015,857	18,055	107,840,567	18,870	5,715	NA
2	Stage I to IV treatment (current)+Trastuzumab (70%)	12,682	156,929,320	2,015,857	18,055	158,963,231	21,645	7,344	NA
3	Stage I treatment+relapse (95%)	2,375	65,738,476	2,514,872	18,958	68,272,306	7,993	8,541	NA
4	Stage II treatment+relapse (95%)	6,815	103,978,996	2,514,872	18,958	106,512,826	10,629	10,021	NA
5	Stage III treatment+relapse (95%)	5,834	87,447,510	2,514,872	18,958	89,981,341	6,015	14,960	NA
6	Stage IV treatment (95%)	2,186	71,452,527	2,514,872	18,958	73,986,358	1,503	49,231	NA
7	Basic Palliative Care (BPC) (95%)	2,186	53,723,979	15,215,345	71,094	69,010,419	1,513	45,609	NA
8	Extended Palliative Care (EPC) (95%)	2,186	101,903,885	16,563,415	71,094	118,538,394	1,523	77,813	NA
9	Stage I to IV treatment combined (current 95%)	17,211	144,702,484	3,698,580	18,958	148,420,023	25,609	5,796	NA
10	Biennial mammography screening (50–70)+treatment of stage I to IV (95%)	17,211	277,083,624	33,291,106	56,875	310,431,605	44,192	7,025	12,718
11	Biennial mammography screening (50–70)+treatment of stage I to IV+Trastuzumab (95%)	17,211	324,996,119	33,291,106	56,875	358,344,101	47,616	7,526	13,994
12	Biennial mammography screening (40–70)+treatment of stage I to IV (95%)	17,211	389,559,667	33,287,097	56,875	422,903,640	50,714	8,339	NA
13	Biennial mammography screening (40–70)+treatment of stage I to IV+Trastuzumab (95%)	17,211	434,231,086	33,287,097	56,875	467,575,059	53,998	8,659	17,115
14	Basic awareness outreach program+Mass-media Awareness Raising (MAR)+treatment of stage I to IV (95%)	17,211	149,330,033	15,890,849	28,438	165,249,320	32,908	5,021	5,021
15	Biennial Clinical Breast Examination (CBE) screening (40–70)+treatment of stage I to IV (95%)	17,211	227,545,334	32,896,957	51,188	260,493,479	39,769	6,550	NA
16	MAR+BPC+treatment of stage I to III (95%)	17,211	184,908,999	29,692,145	99,532	214,700,676	32,919	6,522	NA
17	Biennial CBE Screening+BPC+treatment of stage I to III (95%)	17,211	247,889,383	31,257,913	122,282	279,269,578	39,778	7,021	NA
18	Biennial mammography Screening (40–70)+BPC+treatment stage I to III (95%)	17,211	400,120,033	41,081,697	127,970	441,329,699	50,722	8,701	NA
19	Biennial mammography Screening (50–70)+EPC+treatment of stage I to III (95%)	17,211	296,212,416	41,169,323	127,970	337,509,708	44,210	7,634	NA

a All costs in this table are in 2009 US$ (1MXN = 0,0765697 US$).

b DALYs, disability-adjusted life-years (age weighted, discounted).

c ACER: Average cost-effectiveness ratio compared to the do nothing-scenario (US$ per DALY averted).

d ICER: Incremental cost effectiveness ratio, ratio of additional cost per additional life-year saved when next intervention is added to a mix on the intervention path (additional US$ per additional DALY saved).

### Costa Rica

Table 4 shows the annual number of DALYs averted in treating the individual stages I–IV to vary between 193 (stage III) and 573 (stage II). Jointly these interventions in each stage can avert almost 1,400 DALYs per year. Adding palliative care only averts a small number of DALYs. The costs of treating the individual stages range between approximately US$4 million and US$5 million per year. Adding basic and extensive palliative care programs to stage IV treatment adds approximately US$0.1 and US$1 million to the yearly costs of stage IV treatment. At the 80% coverage level the current country situation in Costa Rica is highly cost-effective with an ICER below the country's GDP per capita, i.e. US$4,739/DALY. In expanding Costa Rica's breast cancer services, our analysis shows that treatment of all stages plus a CBE screening program targeting women between 40 and 70 years of age (I–IV+CBE (40–70)) is the next best option. At a total yearly cost of almost US$13 million, CBE averts 2,381 DALYs per year. This can be considered a very cost-effective intervention as the ICER of this intervention is below one time Costa Rica's GDP per DALY.

From figure 2 it follows that although the ACER of implementing mammography screening for women between 50–70 years is still below Costa Rica's GDP per capita per DALY, the ICER (as compared to CBE screening) is not lower than this threshold (i.e. the slope of the expansion path is steeper than US$6,629/DALY). While still considered a cost-effective intervention, mammography screening in age group 50–69 averts 2,619 DALYs per year at a yearly cost of US$16 million. Increasing the age group for mammography screening to women between 40–70 years shows a similar trend, i.e. averting 3,015 DALYs at an annual cost of US$21 million can be considered cost-effective. Adding Trastuzumab to this intervention, while resulting in the highest number of DALYs averted per year, i.e. 3,274 DALYs at a total yearly cost of US$29 million, is not considered cost-effective as its ICER is above the three times GDP per DALY threshold.

The combinations of various interventions are all close to the expansion path meaning they avert DALYs at a slightly less favorable ICER but could nevertheless be meaningful to implement. For example, expanding the current program's coverage to reach 95% or implementing a Mass-media Awareness Raising program (MAR), could be interesting options if the available budget is not sufficient to implement a screening strategy.

### Mexico

Table 5 shows that the annual number of DALYs averted in the individual stages I–IV varies between 1,503 (stage IV) and 10,629 (stage II). Jointly these interventions in each stage avert approximately 26,000 DALYs per year. The addition of palliative care does not gain much health.

With an ACER of US$5,715 the current situation with 70% coverage is very cost-effective. The analysis shows it is better to increase the coverage level of the current intervention to 95% instead of adding Trastuzumab. In our analysis, implementing a program of Mass-media awareness raising (MAR) buys health most efficiently. Our results show that MAR averts 32,908 DALYs per year at a yearly cost of US$165 million, which leads to an ACER of US$5,021 per DALY averted. When a higher budget would be available, implementing mammography screening for women aged 50–70 would be the first next step. This intervention averts 44,192 DALYs per year at an estimated yearly cost of US$310 million. Even more resources would allow to subsequently add Trastuzumab and increase the age group to 40–70. These interventions fill out the expansion path and avert 47,616 and 50,714 DALYs per year at an estimated yearly cost of US$358 and US$471 million respectively. It should be noted that a CBE screening program, with an expected health gain of 39,769 DALYs averted at a cost of US$260 million, could be an interesting ‘in-between’ option.

### Sensitivity analysis

Sensitivity analysis showed our results to be particularly sensitive to different assumptions on stage distribution at presentation and case fatality rates (tables 6 and 7). The Costa Rican CFs we obtained from Ortiz [24] differed strongly from those we deem to reflect technical efficiency [7], [22]. Using these CFs causes the ACERs to vary between minus 82.7% for stage I and plus 65.5% for stage II. With regards to the current stage distribution, for Costa Rica we used the distribution from Groot et al. [7]. With this less favorable stage distribution, the current country situation was not part of the expansion path anymore. Rather, the CBE screening program now became the most cost-effective.

**Table 6:** Costa Rica - Results of sensitivity analysis on average cost-effectiveness ratio (ACER).

#	Intervention scenarios	ACER	Alternative stage distribution^a^	Case fatality rates^b^	Disability weights +10%	Costs outpatient visits +25%	Costs mammo-graphy +200%	Costs mastec-tomy Mexico	Costs lumpec-tomy Mexico	Capacity utilization equipment −25%^c^	Sensitivity of CBE and mammography −25%^d^	Attendance rates screening program 60%
**1**	Current country specific situation 80%	4,739	5,519	4,447	5,132	4,882	6,218	4,931	4,901	4,739		
**2**	Stage I to IV treatment combined (current 80%)+Trastuzumab	9,180	9,838	8,226	9,796	9,325	10,402	9,337	9,313	9,180		
**3**	Stage I treatment	9,218	11,569	53,348	13,846	9,690	13,096	9,308	9,340	9,218		
**4**	Stage II treatment	9,007	16,395	5,442	9,605	9,369	12,032	9,183	9,250	9,007		
**5**	Stage III treatment	24,587	7,630	19,686	26,352	25,608	33,092	25,133	24,709	24,587		
**6**	Stage IV treatment	24,559	29,195	25,869	26,307	25,774	33,715	24,646	24,559	24,559		
**7**	Basic Palliative Care (BPC)	25,078	30,875	26,412	26,833	26,248	34,179	25,121	25,078	25,078		
**8**	Extended Palliative Care (EPC)	31,852	38,068	33,542	34,044	33,245	40,897	31,895	31,852	31,852		
**9**	Stage I to IV treatment combined (current 95%)	5,417	6,254	5,082	5,866	5,592	6,895	5,609	5,579	5,417		
**10**	Biennial mammography screening (50–70 years)+Stage I to IV treatment	6,228	4,464	7,060	6,565	6,538	10,589	6,336	6,330	6,228	7,535	7,723
**11**	Biennial mammography screening (50–70 years)+Stage I to IV treatment+Trastuzumab	8,402	6,251	9,013	8,807	8,684	12,365	8,501	8,495	8,402	9,856	10,058
**12**	Biennial mammography screening (40–70 years)+Stage I to IV treatment	7,085	5,216	8,069	7,433	7,496	13,203	7,174	7,182	7,085	7,677	8,114
**13**	Biennial mammography screening (40–70 years)+Stage I to IV treatment+Trastuzumab	8,924	6,769	9,674	9,322	9,303	14,562	9,006	9,014	8,924	9,566	10,031
**14**	Mass media awareness raising (MAR)+treatment of stage I to IV	5,857	3,965	5,947	6,247	6,017	7,232	6,010	5,987	5,857		
**15**	Biennial clinical breast examination (CBE) screening (40–69)+treatment of stage I to IV	5,397	3,794	6,095	5,710	5,916	5,977	5,520	5,503	5,397	6,881	7,028
**16**	MAR+BPC+Stage I to III treatment	6,044	4,092	6,137	6,446	6,206	7,418	6,195	6,174	6,044		
**17**	Biennial CBE screening (40–69)+BPC+treatment of stage I to III	5,415	3,806	6,115	5,728	5,934	5,994	5,537	5,520	5,415	6,919	7,068
**18**	Biennial mammography screening (40–69)+BPC+treatment of stage I to III	7,229	5,323	8,232	7,583	7,641	13,345	7,318	7,326	7,229	7,836	8,284
**19**	Biennial mammography screening (50–69)+EPC+treatment of stage I to III	6,444	4,619	7,304	6,792	6,756	10,803	6,551	6,545	6,444	7,815	8,013

a Alternative stage distribution: 9.4% stage I, 14.2% stage II, 58.0% stage III, 18.4% stage IV [7].

b Alternative Case Fatality rates; 0,0174 stage I, 0,0284 stage II, 0,0832 stage III, 0,2855 stage IV [24].

c Mechanical equipment (e.g. mammography machines, CT, X-ray).

d Alternative assumptions on effectiveness of awareness interventions (−25%), sensitivity of CBE, and stage shifts of CBE screening.

**Table 7:** Mexico- Results of sensitivity analysis on average cost-effectiveness ratio (ACER).

#	Intervention scenarios	ACER	Alternative stage distribution^a^	Alternative stage distribution^b^	Alternative stage distribution^c^	Case fatality rates^d^	Disability weights +10%	Costs outpatient visits +25%	Costs mammo-graphy +200%	Capacity utilization equipment −25%^e^	Sensitivity of CBE and mammography −25%^f^	Attendance rates screening program 60%
**1**	Current country specific situation 70%	5,715	6,081	6,576	5,742	7,696	7,764	5,865	6,861	5,713		
**2**	Stage I to IV treatment combined (current 70%)+Trastuzumab	7,344	7,405	7,330	7,513	9,031	8,768	7,482	8,400	7,342		
**3**	Stage I treatment	8,541	11,835	11,407	9,745	19,263	11,997	8,933	11,407	8,534		
**4**	Stage II treatment	10,021	9,613	9,026	16,334	11,433	14,721	10,326	12,416	10,014		
**5**	Stage III treatment	14,960	12,661	15,139	9,786	18,509	31,038	15,515	19,071	14,950		
**6**	Stage IV treatment	49,231	55,817	169,157	37,773	46,698	52,548	51,336	63,668	49,192		
**7**	Basic Palliative Care (BPC)	45,609	53,896	195,026	31,995	43,268	48,621	47,661	59,946	45,569		
**8**	Extended Palliative Care (EPC)	77,813	85,844	229,906	62,358	73,858	82,886	80,085	92,056	77,774		
**9**	Stage I to IV treatment combined (current 95%)	5,796	6,168	6,673	5,820	7,804	7,874	5,946	6,942	5,793		
**10**	Biennial mammography screening (50–70 years)+Stage I to IV treatment	7,025	5,703	8,161	4,043	9,059	7,649	7,397	11,541	7,023	10,041	10,567
**11**	Biennial mammography screening (50–70 years)+Stage I to IV treatment+Trastuzumab	7,526	6,261	8,495	4,607	9,462	8,108	7,526	7,526	7,526	10,051	10,460
**12**	Biennial mammography screening (40–70 years)+Stage I to IV treatment	8,339	6,992	9,425	5,169	10,572	8,945	8,863	15,109	8,338	9,525	10,509
**13**	Biennial mammography screening (40–70 years)+Stage I to IV treatment+Trastuzumab	8,659	7,377	9,599	5,602	10,859	9,226	9,148	14,974	8,658	9,821	10,688
**14**	Mass media awareness raising (MAR)+treatment of stage I to IV	5,021	3,656	6,503	2,293	6,604	5,799	5,172	6,186	5,019		
**15**	Biennial clinical breast examination (CBE) screening (40–69)+treatment of stage I to IV	6,550	5,149	7,837	3,510	8,579	7,246	7,218	7,097	6,549	11,097	11,711
**16**	MAR+BPC+Stage I to III treatment	6,522	4,751	8,452	2,981	8,661	7,531	6,671	7,613	6,520		
**17**	Biennial CBE screening (40–69)+BPC+treatment of stage I to III	7,021	5,519	8,402	3,763	9,195	7,766	7,690	7,568	7,019	12,194	12,893
**18**	Biennial mammography screening (40–69)+BPC+treatment of stage I to III	8,701	7,296	9,836	5,394	11,023	9,333	9,226	15,490	8,700	10,010	11,103
**19**	Biennial mammography screening (50–69)+EPC+treatment of stage I to III	7,634	6,200	8,874	4,395	9,844	8,312	8,009	12,149	7,633	11,152	11,765

a Unidad de Análisis Económico - 8.4% stage I, 38.5% stage II, 42.5% stage III, 10.6% stage IV [42].

b 9.7% stage I, 52.7% stage II, 34.8% stage III, 2.8% stage IV [41].

c 9.4% stage I, 14.2% stage II, 58.0% stage III, 18.4% stage IV [7].

d Alternative Case Fatality rates: 0,013 stage I, 0,042 stage II, 0,102 stage III, 0,266 stage IV [35].

e Mechanical equipment (e.g. mammography machines, CT, X-ray).

f Alternative assumptions on effectiveness of awareness interventions (−25%), sensitivity of CBE, and stage shifts of CBE screening.

For Mexico we ran the model with three different current stage distributions obtained from different studies [7], [41], [42]. These different stage distributions caused the ACERs to increase between 0–15%. When using the higher CFs from Salomon et al. [35] for the intervention scenarios, the ACERs increased to a larger extent (34.7% for the current country situation).

For both countries, changes in the other parameters also led to different outcomes although their impact was smaller. For example, in Costa Rica the WHO default unit costs for a mastectomy or a lumpectomy were relatively low. Unable to obtain these unit costs from Costa Rica, using the higher Mexican unit costs showed their impact on the ACERs to be marginal.

## Discussion

Our results indicate that in both Costa Rica and Mexico treating stage IV disease only, or treating stage IV and providing basic or extended palliative care is not cost-effective. In general, interventions ensuring more patients to present at the hospital in earlier stages seem the most cost-effective.

These results are in line with other studies which find mammography screening for women aged 50–70 to be cost-effective in sub-Saharan Africa and South East Asia [7], [43]. Although Ginsberg et al. did not study the cost-effectiveness of clinical breast examination or other awareness raising programs, they acknowledge less expensive means of early detection in limited resource settings could be cost-effective in LMICs [43]. When modeling the expected outcomes of such strategies - though based on limited evidence - Zelle et al. find that CBE screening or mass media awareness raising interventions seem indeed cost-effective in Ghana [22].

Although mammography interventions can be considered cost-effective, their total annual costs (budget impact) are high and may therefore not be appropriate for wide scale implementation.

If the necessary resources are not available both countries could choose to lower coverage levels or implement interventions with comparable ACERs (buying health just as efficiently) but with lower budget impact. For Costa Rica, our analysis shows the most cost-effective option for expanding the current breast cancer services would be a CBE screening program combined with treatment of all stages. The yearly costs of this program are about US$12 million. In 2009, the per capita health expenditure in Costa Rica was US$660 (10.3% of total GDP) [37]. With a population of approximately 4.5 million, implementing a CBE screening program would add US$2.82 to this amount (0.43% increase). Although this increase may seem feasible, the implementation and effectiveness of this program is highly dependent on the availability of human resources and the capacity of the healthcare system to refer and treat all new-found cases [44]–[46]. Also, if the implementation of a CBE screening program would be unfeasible, MAR could be an interesting option as it is slightly less cost-effective but has a smaller yearly budget impact (US$10 million). Yet, the very limited evidence on MAR's effectiveness requires our estimates to be interpreted with caution. Implementing a screening program for which the evidence base is stronger (e.g. mammography for women between 50–70 years of age) could be recommended if the yearly costs of US$16 million are affordable. Mammography screening in age group 40–70 costs much more (about US$21 million) and is therefore less economically attractive.

The Mexican MoH already decided to start increasing the use of the available infrastructure and mammography equipment for the population most at risk (women 50 to 70 years old and women with more than two risk factors). The gradual expansion will give enough time to train the required human resources. From our analysis the yearly costs of a mammography screening program for women 50–70 years of age at 95% coverage eventually would be US$310 million per year, a threefold increase over the current scenario. Next, once a reasonable increase on coverage would be reached the Mexican MoH plans to increase the coverage rate to women between 40–49 years of age [47]. According to our estimates the yearly costs of implementing such a program would be US$422 million. With approximately 110 million inhabitants and a per capita health expenditure of US$525 in 2009 (6.43% of total GDP) [37], implementing these programs would add US$2.82 (0.54% increase) and US$3.84 (0.72% increase) respectively to per capita health expenditure.

However, our analysis shows perhaps that strengthening actual MAR or CBE screening programs to be a more attractive first step in improving breast cancer services from an economic perspective. With yearly costs of US$165 and US$260 million if started from zero, the strengthening of existing programs is more affordable and more politically feasible as it would represent modest increases to existing budgets.

One of the principal questions we received from policy makers in both Costa Rica and Mexico concerned the addition of Trastuzumab to the treatment regimens. In Costa Rica we assumed 30% of the breast cancer patients have overexpression of the HER2/neu+ gene and are eligible for Trastuzumab [48]. As a result of adding Trastuzumab, in Costa Rica between 230–270 extra DALYs/year are averted at an additional cost of approximately US$7 million per year. For Mexico we obtained the actual proportion of patients receiving Trastuzumab in IMSS. Here the health gains comprise between 2,800 and 3,400 extra DALYs/year averted and the additional costs fall between US$45–51 million. It is worth noting that in Mexico Trastuzumab is already provided as part of the treatment for all eligible women in stages I to IV. Our analysis shows the addition of this bio-pharmaceutical to increase the cost of treatment of stages I to IV by more than 48%, generating the need of developing public policies focused on negotiating price reductions that can contribute to the mid- and long-term financial sustainability. The use of tools as the ones presented in this paper can provide technical evidence on the benchmark price that the Mexican health system could use in negotiations considering the threshold of one times the GDP per capita.

The limitations regarding the model are essentially the same as those reported in previous studies [7], [22]. First, as evidence on the effectiveness of awareness raising, CBE and mammography screening in Costa Rica and Mexico were absent, we relied on the same model approach as used by Zelle et al. [22]. Second, when calculating unit costs for Mexico we did not account for the mark up of transportation costs (as generally recommended by WHO-CHOICE) and did not include the costs of facilities. Including these costs would have probably resulted in slightly higher unit costs. Third, in adopting a health care perspective we did not take into account travel and opportunity costs. Including these costs would probably have increased costs generally. Fourth, we did not carry out a probabilistic sensitivity analysis. Carrying out such analysis would have shown worse ACERs when parameters are jointly changed in the negative direction (i.e. higher CFs and costs/worse stage distribution). Nonetheless, our deterministic sensitivity analysis shows the direction in which ACERs would change is clear and our general conclusions remain the same although the ranges of several ACERs are overlapping. The limitations fit within the overall goal of WHO-CHOICE which is to provide general indications of cost-effectiveness, i.e. not precise estimates of specific interventions.

In summary, for improving their current breast cancer control programs, our analysis suggests that both Costa Rica and Mexico would benefit from implementing strategies that advance early detection. For these countries, a mass-media awareness raising program and/or a CBE screening program coupled with treatment of all stages and careful monitoring and evaluation could be feasible options. If these strategies are implemented, the provision of breast cancer diagnostic, referral, treatment and, when possible, basic palliative care services is essential and should be facilitated simultaneously.
